# Transient Absorption Spectroscopy of Films: Impact of Refractive Index

**DOI:** 10.1021/acs.jpcc.4c00981

**Published:** 2024-04-05

**Authors:** Hannu P. Pasanen, Ramsha Khan, Jokotadeola A. Odutola, Nikolai V. Tkachenko

**Affiliations:** † Ultrafast Dynamics Group Physical Science and Engineering Division, King Abdullah University of Science and Technology, Thuwal 4700, Kingdom of Saudi Arabia; ‡ Chemistry and Advanced Materials Group Faculty of Engineering and Natural Sciences, Tampere University, Tampere 33014, Finland

## Abstract

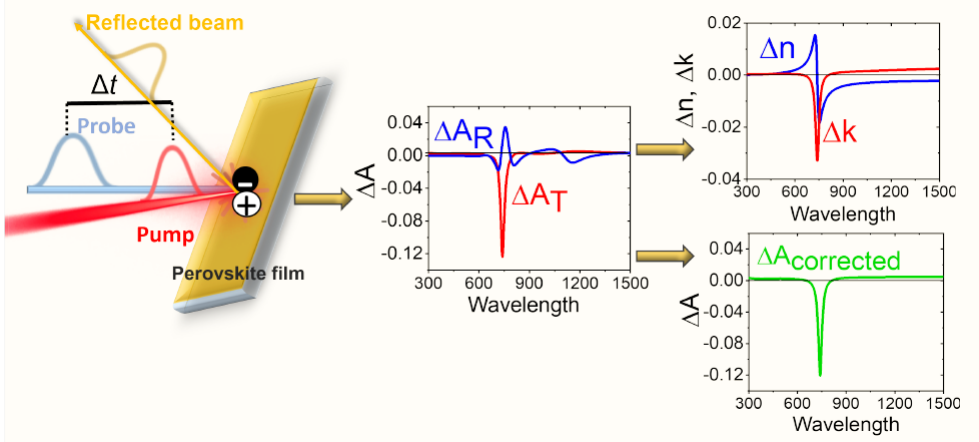

Transient absorption spectroscopy is a powerful technique to study the photoinduced phenomena in a wide range of states from solutions to solid film samples. It was designed and developed based on photoinduced absorption changes or that photoexcitation triggers a chain of reactions with intermediate states or reaction steps with presumably different absorption spectra. However, according to general electromagnetic theory, any change in the absorption properties of a medium is accompanied by a change in the refractive properties. Although this photoinduced change in refractive index has a negligible effect on solution measurements, it may significantly affect the measured response of thin films. In this Perspective paper, we examine why and how the measured responses of films differ from their expected “pure” absorption responses. The effect of photoinduced refractive index change can be concluded and studied by comparing the transmitted and reflected probe light responses. Another discussed aspect is the effect of light interference on thin films. Finally, new opportunities of monitoring the photocarrier migration in films and studying nontransparent samples using the reflected probe light response are discussed. Most of the examples provided in this article focus on studies involving perovskite, TiO_2_, and graphene-based films, but the general discussion and conclusions can be applicable to a wide range of semiconductor and thin metallic films.

## Introduction

Transient absorption spectroscopy (TAS) is an indispensable, widely used, and well-developed technique to study fast and ultrafast photoinduced phenomena in a wide range of materials. It is a tool providing superior time resolution down to femtoseconds to deliver key information on the dynamics of photochemistry reactions^1,2^ and primary events in natural photosynthesis,^3^ and recently it is one of the advanced techniques to study and optimize materials developed for solar cell and photocatalysis applications.^4−7^

An important part of TAS research is data analysis, which allows us to identify photoreaction intermediate states and obtain quantitative characteristics associated with the reaction kinetics.^1,3,5−7^ Typical TAS measurements can be presented as two-dimensional arrays of type △*A*(*t*, λ) which shows how the sample transient absorption (TA) response △*A* depends on the delay time *t* and the monitoring (probe) wavelength λ. It is assumed that the TA is proportional to population of the intermediate states^1,8^
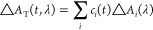
1where *c*_*i*_(*t*) and △*A*_*i*_(λ) are the concentration at delay time *t* and the differential absorbance at wavelength λ of intermediate state *i*, respectively. It can be shown that this assumption is valid for solution measurements, which can also be applied to solid-state samples in many cases, although not always.

Generally, the absorption and refractive properties of a medium are linked to each other.^9,10^ If one changes, then the other changes too. However, in a solution of dye molecules or alike, the absorption properties of the sample are determined by the dye, but the refractive properties are dominated by the solvent molecules. Consequently, the change in the sample’s refractive properties upon the dye’s photoexcitation event can be neglected. However, this is not necessarily the case for condensed matter, such as molecular or semiconductor films, in which the density of “excited species” is large enough such that the change in refractive properties may have a significant effect on the sample TA response.^11−14^

In this Perspective paper, we examine the cases whereby a photoinduced refractive index change has a significant impact on the observable TA response. This must be taken into account before solely applying the data analysis based on absorption changes, i.e., eq 1, as ignoring the refractive index changes may lead to erroneous interpretation of the results.^15^ Moreover, this improved approach for the analysis of optical properties of the studied films not only avoids misinterpretation but also provides a more complete understanding of the photoinduced reactions, including processes which have no impact on the sample absorption such as carrier diffusion across the film.

## Optical Properties of Films

As a simple example, one can consider a photoactive film, such as a perovskite layer, which experiences a change in refractive index upon photoexcitation. This refractive index change leads to a change in the amount of light reflected from the film surface and consequently alters the amount of transmitted light, including wavelengths, where the sample absorption does not change. As a result, the TA response deviates from that expected for the “pure” absorption change. This was experimentally observed by a few research teams^12,13,16,17^ and even called an “artifact” by some authors.^18^

It can be noted that pure photoinduced changes in the refractive index have opposite effects on the sample reflectance (*R*) and transmittance (*T*). If the refractive index increases, then *R* increases and *T* decreases, but the sum, *T* + *R*, does not change. Whereas, for pure absorption changes, both *R* and *T* change in the same direction; i.e., if sample absorption increases, both *R* and *T* decrease. In addition, light reflected from both surfaces of the film can interfere, which further complicates the transient response^12,19,20^ and will be discussed below.

### Dielectric Function and Drude-Lorentz Model

Within the Maxwell electromagnetic theory, the dielectric properties of a medium are described by the complex dielectric function ε = ε′ + *iε*″, where the real part, ε′, determines the electromagnetic wave propagation velocity in the medium, and the imaginary part, ε″, determines the attenuation of the electromagnetic wave.^9^ In optical calculations, a corresponding parameter is the complex refractive index, *ñ* = *n* + *ik*, where *n* is the real refractive index and *k* is the optical extinction coefficient which is connected to the medium absorption coefficient as α = 4π*k*/λ, where λ is the wavelength. The relation between ε and ñ is ,^17^ which means that *n*^2^ – *k*^2^ = ε′ and 2*nk* = ε″. For most semitransparent materials, *n* ≫ *k*, and thus *n*^2^ ≈ ε′. Since *n* is a “slow” function of λ (*n* ≈ const), *k* is roughly proportional to ε″ (*k* = ε″/2*n*).

The effect of photoinduced changes on the (complex) refractive index can be demonstrated from a very general perspective using perovskite film as an example. The complex refractive index of some perovskite materials have been measured.^21^ However, these values cannot be used directly, if phenomena, such as photoinduced changes in the conduction band (CB) carrier population, are of interest. To approach the problem, one can use the Drude-Lorentz (D-L) model of the dielectric function.^22,23^ The model can include a few Lorentz bands which correspond to transitions between two energy states and a Drude component to model “free” electrons in the CB or so-called plasmon band. Mathematically it can be expressed as
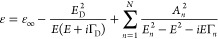
2where *E* = *hν* is the photon energy, *E*_D_ and Γ_D_ are the energy and damping of the Drude component, *A*_*n*_, *E*_*n*_, and Γ_*n*_ are the amplitude, energy, and damping of the Lorentz bands, and ε_*∞*_ is the so-called high-frequency dielectric constant which accounts for the effect of high-energy transitions outside of the frequency (wavelength) range of interest. Lim et al. demonstrated the use of the Drude component to estimate charge carrier mobility in lead halide perovskite thin films from the photoinduced change in reflectance.^24^

Modeling of the perovskite absorption spectrum in the visible–near-IR range (300–1 500 nm) requires at least three Lorentz bands (*N* = 3) at roughly 350, 560, and 740 nm. For the sake of simplicity, we assume that there are no free carriers in a nonexcited perovskite film and model the ground state as the sum of three Lorentz bands only. The Drude component was added only to model the excited state.

**Figure 1:**
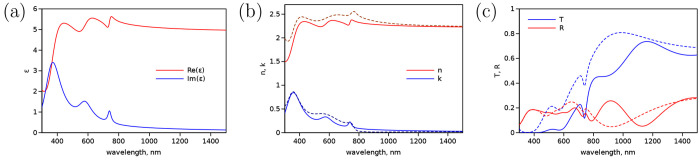
Model spectra of (a) real and imaginary parts of dielectric function ε, (b) *n* and *k*, and (c) transmittance (*T*) and reflectance (*R*) spectra of 300 nm (dashed line) and 500 nm (solid line) thick perovskite film. The dashed lines in (b) show experimental *n* and *k* spectra of perovskite film.^21^

It can be noted that the Lorentz band is not ideal for the modeling and Gaussian bands would be a better fit. However, the “theoretical” advantage of the Lorentz band is that it complies with the Kramers–Kronig (KK) relation, linking real and imaginary parts of the dielectric function, meaning that both real and imaginary parts can be calculated from eq 2. If a Gaussian band is used to model ε″, which determines the medium absorption, ε′ has to be calculated using a KK integral calculated ideally in an infinite frequency range,^17^ which imposes some practical complications as discussed below. Therefore, Lorentz bands are used here for simulations to obtain consistent qualitative models while not assuming an exact model for the perovskite films. The modeled spectra are presented in Figure 1. The parameters were adjusted to attain reasonable qualitative agreement between the model and measured spectra of *n* and *k* (Figure 1b), and the Lorentz band parameters are listed in Table 1.

**Table 1:** Lorentz Band Parameters Used to Model the Perovskite Films^a^

*n*	1	2	3
*E*_*n*_, nm	740	580	365
*A*_*n*_, eV	0.24	0.87	3.7
Γ_*n*_, eV	0.06	0.4	1.2

a See Figure 1.

### Effect of Light Interference in the Film

If film thickness is comparable with the monitoring wavelength, the interference of light reflected from the front and back surfaces affects the film transmittance and reflectance and has to be accounted for. This phenomenon is known as Fabry–Perot interference, typically discussed in terms of constructive and destructive interference, and results in wavy shapes of *T* and *R* spectra. The interference pattern depends on the film thickness, *d*, and it can be observed already at *d* ≈ λ/10 or tens of nanometers in the optical wavelength range, which is roughly the lower limit. As the thickness of film increases, the period of waves in the spectra decreases, but depending on the spectrum resolution, it can still be observed at *d* ≈ 100λ if the spectrum resolution is △λ < λ^2^/2*dn* or, roughly, 1 nm in the optical range.

The *n* and *k* spectra can be used to calculate *T*, *R*, and absorbance (*A*) spectra of a film with a known thickness, *d*. Moreover, the interference of the light reflected at interfaces of multilayer film can be accounted for using a well-developed transfer matrix method (TMM).^19,25,26^ To illustrate the effect of interference on the measured spectra, we assume that the film is deposited on a quartz substrate with a known refractive index spectrum.^27^ The calculations carried out for two thicknesses, *d* = 300 and 500 nm, are presented in Figure 1c. The light interference pattern can be noticed in the near-IR range (λ > 750 nm), where the perovskite itself has no absorption but both *T* and *R* spectra have apparent spectral features. This is a well-known property of films reported and observed for perovskite films, in particular.^12,16^

Another D-L application is to model the *T* and *R* spectra for thin conducting films such as undoped^22^ and doped^28^ multilayer graphene-based films. In this case, the Drude part corresponds to the free carrier response while one Lorentz band is used to model the π → π* transition in the UV region at 4.6 eV. This has been shown as an accurate model for undoped films from 360–1100 nm.^22^ In a study with doped films,^28^ a common set of D-L parameters was calculated from the measured *T* and *R* spectra for films while using the TMM^19,25,26^ to account for different optical thicknesses.

Similarly, the D-L model can interpret optoelectronic changes in other conductive thin films such as tin-doped indium oxide (ITO) films using *T* and *R* spectra.^29^ In this manner, changes in conductivity with different silver concentrations and annealing temperatures are explained by the *n* and *k* terms. The key difference was the use of ellipsometry to determine refractive index changes and account for interference effects in the *T* and *R* spectra.

## Transient Absorption Response

**Figure 2:**
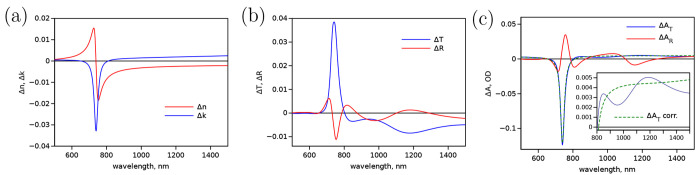
Model differential spectra of the perovskite excited state calculated for (a) △*n* and △*k*, (b) transmittance (△*T*) and reflectance (△*R*), and (c) corresponding absorbance change for standard transient transmittance △*A*_T_ and reflectance △*A*_R_ measurements. The green dashed line in plot (c), △*A*_T_ corr., represents the △*A*_T_ calculated using eq 4, see text for details.

In the excited state and after thermal relaxation, the carriers from the top of the valence band (VB) are promoted to the bottom of the CB, which reduces the absorption at the lowest energy band, *A*_1_, in the case of the model we used above for perovskite film (Figure 1, Table 1). The carriers at the CB are “free” carriers, and their optical properties are described by the Drude component.^30^ Thus, to model the excited state, we reduce *A*_1_ by a small value and add a small Drude component. To make the modeling reasonably close to the experimental results, the ratio between the decrease of *A*_1_ and the added Drude amplitude *E*_D_ was adjusted to generate spectra △*n*(λ) and △*k*(λ) similar to those reported for the 500 nm perovskite film.^12^ The modeled △*n*(λ) and △*k*(λ) are presented in Figure 2a. Then, TMM was used to calculate △*T* and △*R*, as shown in Figure 2b. Typically, TAS instruments recalculate measured signals to transient absorbance, which is △*A*_T_ = – log_10_(1 + △*T*/*T*) for standard transmittance measurements and △*A*_R_ = – log_10_(1 + △*R*/*R*) for reflectance measurements. Thus, calculated model responses are shown in Figure 2c.

Apparently, the interpretation of the “photo-induced” change in the complex refractive index  is straightforward: there is a “bleach” of the lowest energy band (△*k* at 740 nm) and a broad band absorption increasing in intensity toward the longer wavelengths due to the free carriers in the CB. However, the interpretation of the measured transmittance (△*T*) and reflectance (△*R*) changes (or corresponding absorbance values △*A*_T_ and △*A*_R_) is more challenging. The bleaching of the lower energy band is the most clear and distinct feature of the △*A*_T_ response, and it is widely used to monitor dynamics of photocarriers in perovskite films,^31^ semiconductors,^32^ and molecular films.^33^ In the latter case, the effect of free carriers may be negligible or may have a very minor impact. Therefore, the TA measurements of molecular films are interpreted in a manner similar to that of the solutions. Then, the negative △*A*_T_ is termed ground-state bleaching (GSB) and used as an indicator that the molecules are not in their ground state.

Outside to this main feature, there is a broad band response due to free carriers (Drude component) with a wavy shape.^12,34^ These waves resemble those of steady-state *T* and *R* spectra in Figure 1c at wavelengths longer than the band gap (λ ≥ 800 nm) and have the same origin: the light thin-film interference (TFI). The photoinduced change of the refractive index affects the interference pattern and changes the proportion in reflected and transmitted probe light. It can be noted that both △*n* and △*k* have virtually flat spectra with no features at λ > 900 nm (Figure 2a), but the measured △*A*_T_ shows a “fake” band at 1200 nm (inset in Figure 2c). The position of the band would change if identical samples with different thicknesses would be measured. The calculations were done assuming some photoinduced absorption at λ > 900 nm (△*k* ≈ 0.0015). If △*k* = 0, the wavy shape of the measured △*A*_T_ will be shifted down to an average value of △*A*_T_ being zero, which results in a negative △*A*_T_ around wavelengths 1000 and 1500 nm despite the fact that the ground-state *A*_T_ = 0 and it cannot decrease. This “fake” response was observed in perovskite and TiO_2_ films^12,35,36^ and is discussed below. This highlights the importance of understanding the effects of refractive index changes and light interference in films and finding corrections for measured △*A*_T_ and △*A*_R_ before assigning spectral features to physical processes in the film.

### An Approximation to Measure Absorbance

Formally, the amount of light absorbed by the sample is the difference between the intensity of the incident monitoring beam and the sum of intensities of the reflected and transmitted beams. Neglecting the possible many-pass probe propagation due to the light reflectance at the interfaces, the relation between the light absorbance in the film *A*, the film transmittance *T*, and reflectance *R* is *T* = (1 – *R*)10^–*A*^, where all reflectance is attributed to the front surface and reflectance from the back surface is neglected. Photoexcitation induces changes in all the three values, *T*, *R*, and *A* as
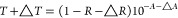
3

If the value of interest is △*A* only, then substituting △*A*_T_ and △*A*_R_ instead of △*T* and △*R*, respectively, one can obtain^24,36^
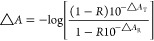
4

The spectrum calculated according to this equation is shown in Figure 2c by the green dashed line (△*A* corr.). The spectrum is free of the wavy shape caused by the probe light interference in the sample and can be analyzed as a standard transient absorbance change with band-like features assigned to transitions between distinct energy states. At least in the case of perovskite films, this approximation (eq 4) solves the problem outside the main bleached band (at λ > 800 nm). In the absence of reflectance, the relation between the transmittance and the absorptance is *T* = exp(−*αd*) = 10^–*A*^; thus, . For the spectra presented in Figure 2c, the band position is at 740 nm and the measured △*A*_T_ = −0.1236, while the corrected value is −0.1207 and “pure” △*A* = −0.1211, or there is no practically significant difference. However, in the range λ = 800 to 1500 nm, the difference is significant as seen from the inset in Figure 2c, but the correction using eq 4 solves the problem.

**Figure 3:**
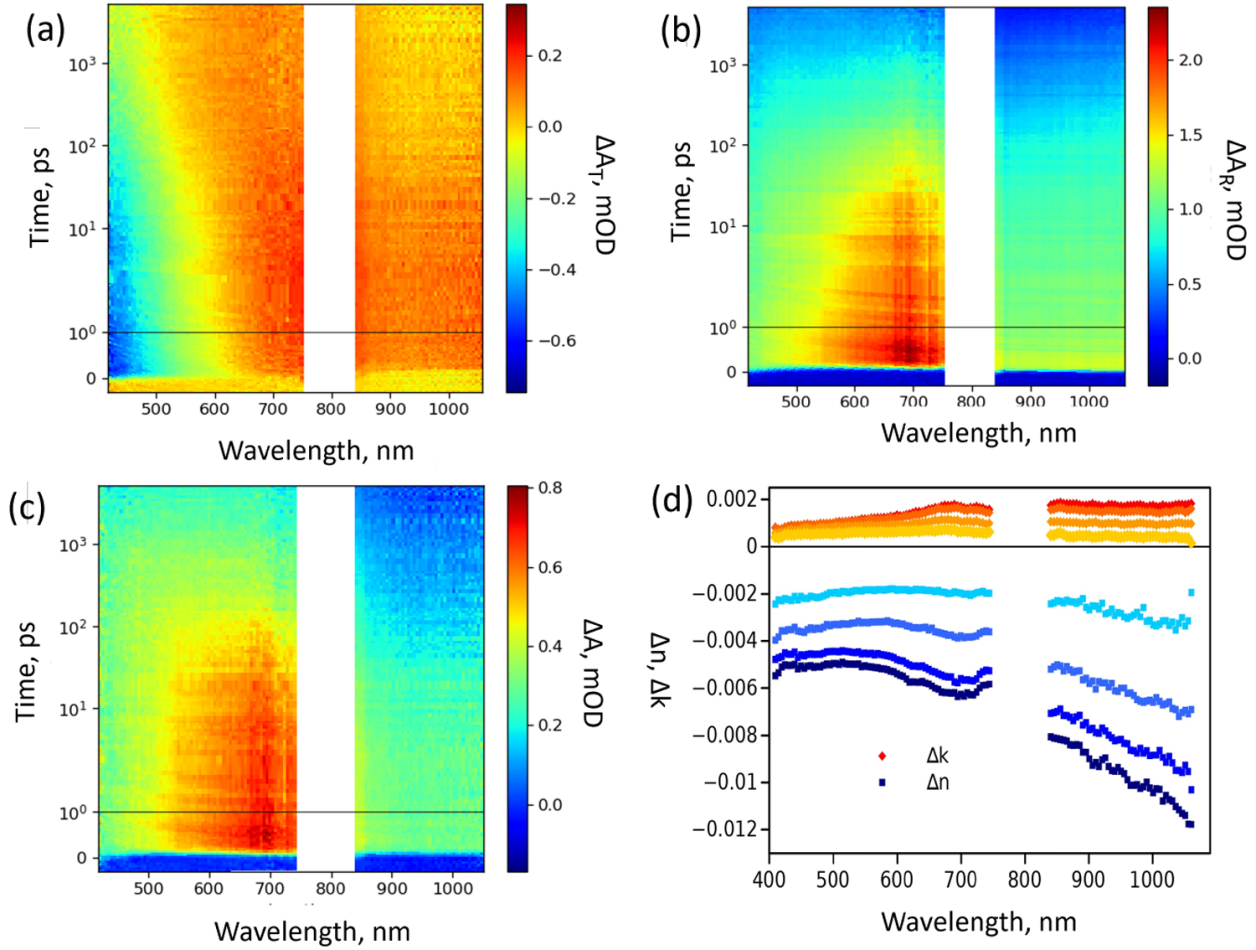
2D presentation of the TA response of 30 nm annealed TiO_2_ thin film deposited via ion beam sputtering excited at 320 nm measured in both (a) transmittance and (b) reflectance modes. (c) Calculated true transient absorbance of the film. The time scale is linear until 1 ps delay time and logarithmic after that, and (d) extracted △*n* and △*k* components at delay times (from darker to lighter) 1, 10, 100, and 1000 ps from the TT and TR spectra. Figure (a–c) reprinted with permission from ref (36). Copyright (2021) Royal Society of Chemistry, and Figure (d) reprinted with permission from ref (39). Copyright (2024) Ramsha Khan.

**Figure 4:**
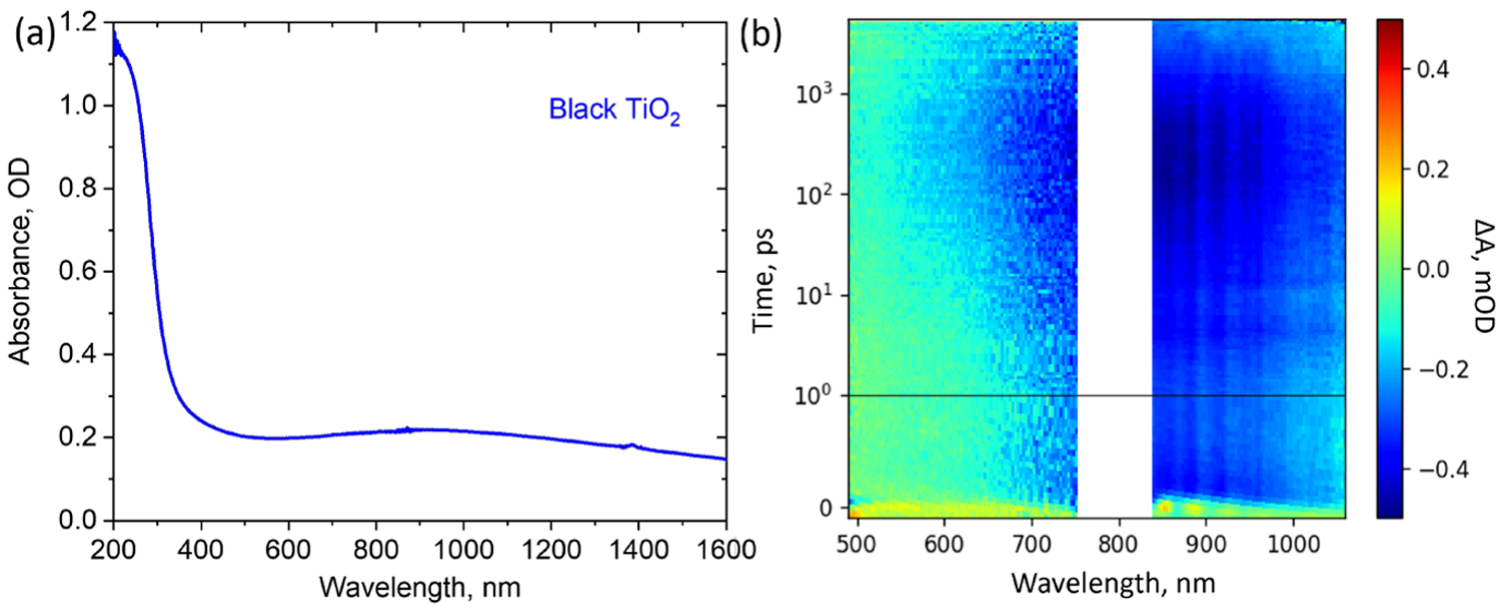
Measured (a) steady-state absorbance spectrum and (b) 2D presentation of the TA response obtained from 320 nm excitation of as-deposited 30 nm TiO_2_ thin film deposited via atomic layer deposition. Reprinted with permission from ref (37). Copyright (2022) American Chemical Society.

Within this approximation, the “true” absorbance change can be estimated from the measured steady-state reflectance spectrum, *R*, and TA measurements carried out in transmittance, △*A*_T_, and reflectance, △*A*_R_, modes. In order to understand the photodynamics of TiO_2_ nanofibers deposited on quartz substrate, Saha et al. utilized TA spectroscopy and found a negative transient response in the visible region and positive broad transient absorption in the IR region.^35^ However, pure TiO_2_ has no visible light absorbance, and hence the negative TA response cannot be ascribed to the bleaching of the ground-state absorption upon photoexcitation. Therefore, this phenomenon was termed as an instrumental artifact in the study. Khan et al. also observed the same negative TA response in the visible region of 30 nm anatase TiO_2_ thin films (heat-treated at 500 °C) prepared by ion beam sputtering, as shown in Figure 3.^36^ Nevertheless, upon measuring the films in both transient transmittance and reflectance modes, a strong transient reflectance of the films was observed as compared to its transmittance. Therefore, by employing eq 4, the true absorbance changes in the film were observed without any bleach as can be seen in Figure 3c. The long-lived TA response is attributed to trapped electrons and holes. However, when Saari et al. prepared 30 nm amorphous “black TiO_2_ films” from atomic layer deposition at 200 °C, the film had increased number of Ti^3+^ species.^37^ These Ti^3+^ defect states induce visible light absorbance as shown in the steady-state absorbance spectrum in Figure 4a.^38^ Since these TiO_2_ films have actual visible absorbance in this case, the TA spectra show photoinduced bleaching in both visible and IR regions due to electron trapping at the intrinsic in-gap states after the pump pulse.

## Evaluation of △*n* and △*k* (and △ε) from TAS Data

TAS experiments can be carried out in two modes, measuring transmitted probe light change (or in transient transmittance (TT) mode) and measuring reflected probe light change (or in transient reflectance (TR) mode). The measured values are relative changes of light intensities △*T*/*T* and △*R*/*R*, respectively. Since *T* and *R* are available from the steady-state measurements of the sample, △*T* and △*R* can be easily calculated. If the excitation density is constant across the film, the effect of the excitation can be written as a change in optical properties of the film; △*n* and △*k*. Thus, the measured pair of values, △*T* and △*R*, can be used to deliver △*n* and △*k* if the ground-state (non excited) values *n* and *k* are known, since *n* and *k* (and film thickness *d*) determine completely the optical properties of the film. There are well-developed analytical methods to calculate *T* and *R* for known *n* and *k* (and film thickness *d*),^40^ including TMM which allows one to calculate *T* and *R* for multilayer structures.^19,25^ This gives the relation (*n*, *k*) → (*T*, *R*), which can be verified experimentally. Therefore, the data (△*T*, △*R*) analysis can be performed in the following steps.

First, having dependence (*n*, *k*) → (*T*, *R*), the derivatives , , , and  are calculated. It can be done analytically if the analytical dependence is available or numerically by introducing a small variation of *n* and *k*, e.g., analyzing (*n* + △*n*, *k* + △*k*) → (*T* + △*T*, *R* + △*R*) by applying the transfer matrix model. This establishes a linear dependence between (△*n*, △*k*) and (△*T*, △*R*).
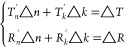
5

Second, the system of linear eq 5 can be solved to calculate (△*n*, △*k*) out of measured values (△*T*, △*R*) and the evaluated derivatives of *T* and *R*. If the spectra △*T*(λ) and △*R*(λ) were measured, then the spectra △*n*(λ) and △*k*(λ) could be calculated within the same range. Pasanen et al. used this approach to study △*n*(λ) and △*k*(λ) of FAMACs perovskite film and obtain refractive index spectra similar to modeling results presented in Figure 2a.^12^

Khan also utilized this approach to separate the △*n*(λ) and △*k*(λ) components of 30 nm anatase TiO_2_ films prepared by ion beam sputtering.^39^ The TT and TR spectra of the films are shown in Figure 3a,b, respectively, and thus by utilizing eq 5, the real and imaginary parts of the complex refractive index are extracted, as shown in Figure 3d. Also, one can notice that the change in △*n*(λ) is larger than that in △*k*(λ) at all studied wavelengths, or △*n* is the main contributor to TT and TR responses.

There is a well-defined relation between *ñ* and ε as was mentioned above . The same approach can be used to calculate (△ε′, △ε″) from the measured values (△*T*, △*R*). This is an especially useful approach if the ground-state dielectric function of the film under study can be described by the D-L model, eq 2, which seems to be the case of graphene mono- and multilayer films.^22^ The obtained (△ε′, △ε″) spectra can be fitted to find out which D-L parameters are the most affected by the photoexcitation.

Odutola et al. applied this method to study pure and doped multilayer graphene-based films.^28^ The doped samples prepared from pyrolysis of chitosan at 900–1 200 °C had unprecedented nanosecond responses termed as the “second wave”. This was in comparison to the well-reported “first wave” sub-picosecond responses present in graphene-based films.^41^

**Figure 5:**
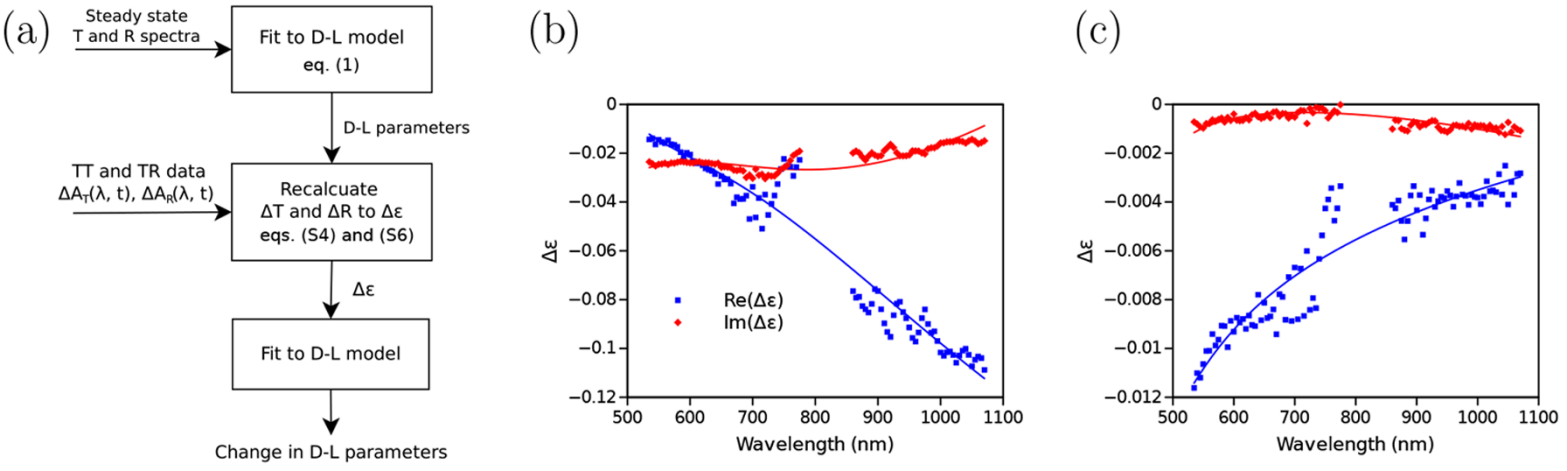
(a) Diagram of graphene TAS data analysis used in ref (28) and obtained dielectric function spectra for (b) sub-picosecond (“first wave”) TAS response and (b) slower nanosecond (“second wave”) response. Reprinted with permission from ref (28). Copyright (2023) American Chemical Society.

To study the samples, the steady-state *T* and *R* spectra were recorded, and TAS measurements were carried out in TT and TR modes, delivering △*A*_T_(λ, *t*) and △*A*_R_(λ, *t*) data, respectively. The analysis began with fitting of the steady-state spectra to establish a suitable D-L model of the sample complex dielectric function ε(λ), as schematically shown in Figure 5a. Then, the aforementioned method was used to calculate (△ε′, △ε″) spectra from the experimental △*A*_T_ and △*A*_R_ for the “first wave” and “second wave” presented in Figure 5b,c.

The spectra for the “first wave” followed the pattern of those from previously reported DFT studies.^42−44^ However, the “second wave” dielectric spectra were opposite compared to those of the “first wave”, which suggested opposing optoelectronic phenomena at different time scales. This was confirmed by fitting the ε spectra to the D-L model, which delivered information on the most affected D-L parameters in the “first wave” and “second wave”. Thus, the free carrier scattering in the “first wave”^45^ was separated from the suspected carrier trapping in the “second wave” which improved catalytic activity of doped films.^46^

### Band Shape Analysis

**Figure 6:**
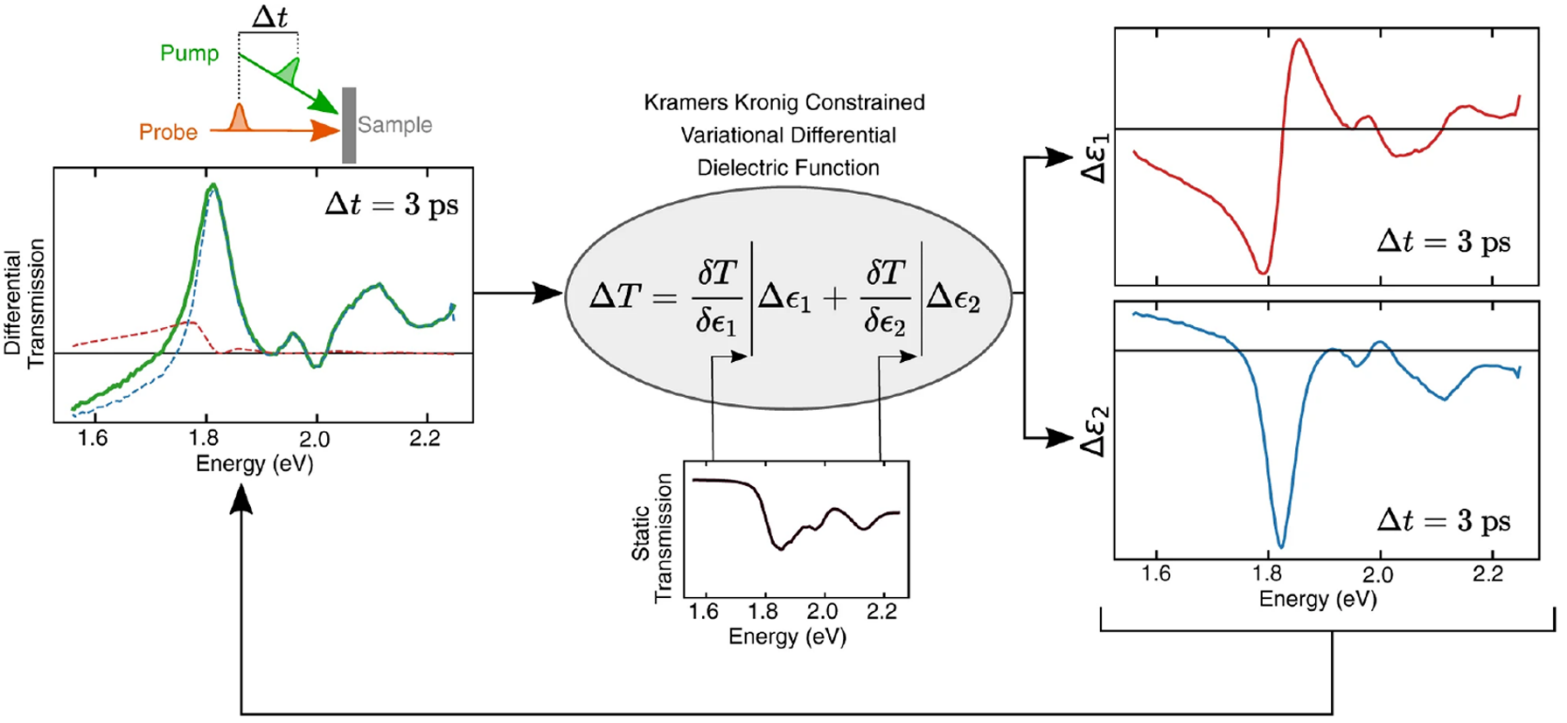
Schematics of the Kramers–Kronig-based analysis technique reported by Ashoka et al. Reprinted with permission from ref (17). Copyright (2010) Springer Nature BV.

As previously discussed, the Lorentz band shape used in the D-L model is often a poor fit to real ε spectra. The Gaussian band or any other shape can be used to model ε″ while ε′ can be calculated using the KK relation (Figure 6). This technique was used by Ashoka et al. to reconstruct △ε′ and △ε″ spectra of perovskite film at different delay times from TAS data.^17^ A similar approach was also used to evaluate refractive index changes of perovskite films under continuous illumination.^17^

Alternatively, KK relations^17,47^ can be employed to calculate the change in refractive index from the change in absorption, or vice versa, for dielectric materials. Although KK relations fundamentally apply only to the dielectric function, and not the complex refractive index, an approximation produces a similar equation for the photoinduced change in refractive index^48−50^
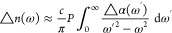
6where *c* is the speed of light, *P* is the Cauchy principal value, α is the absorption coefficient, and ω is the angular frequency. However, the KK relation has certain limitations such as it requires an estimate of △α = 4π△*k*/λ and *P*, and it is not reliable at the edges of the measurement range since it would require information on △α from outside the measurement range. Some studies attributed all changes in sample reflection to photoinduced absorption or bleach,^51,52^ but as shown by eq 6, any change in absorption is accompanied by a change in refractive index at nearby wavelengths.

## Effect of Carrier Distribution and Diffusion

While the interpretation of film TA responses is more complex than that of solutions, the fact that the response is affected by the change in the refractive index opens new opportunities. One of them is the possibility to monitor the change in carrier distribution across the sample.^53−57^ In traditional TA measurements, the photoinduced change of the sample absorbance depends on the total number of the excited states or the photocarriers but does not depend on carrier distribution in the sample.^57^ On the contrary, in films, reflection from the sample surface depends on the refractive index change at the surface (or the surface carrier density) rather than the total number of carriers in the sample. Moreover, the total film reflectance and transmittance are impacted by the gradient in refractive index across the sample, which is induced by photoexcitation and determines the interference pattern inside the sample.^55^

Therefore, although the interference has minor impact on transmittance of samples with high absorption, the sample reflectance can still be strongly affected by the photoinduced gradient of the refractive index close to the reflecting surface.^34^ This gradient changes as carriers move opening the possibility of monitoring carrier diffusion by measuring reflectance and modeling the effect of the carrier gradient on the sample reflectivity.^56^

**Figure 7:**
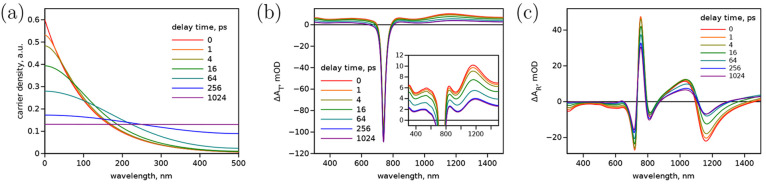
(a) Model change in time of the photocarrier distribution across 500 nm thick perovskite films. Sample absorbance at the excitation wavelength is *A* = 2, and the carrier diffusion coefficient is *D* = 2 cm^2^ s^–1^. Corresponding TA responses for (b) transmitted and (c) reflected signals.

In order to evaluate the effect of carrier distribution change on the film response, we consider a nonhomogeneous photocarrier distribution due to the excitation light decay as it propagates through the film and the following carrier diffusion leading to homogeneous carrier distribution. To simplify modeling we assume that the total number of carriers stays the same. The carrier distribution change with time across the sample can be obtained by solving a one-dimensional diffusion equation.^58−60^ The results of modeling for *d* = 500 nm film with diffusion coefficient *D* = 2 cm^2^ s^–1^ is presented in Figure 7a. Here we assume the film optical density at the excitation wavelength to be 2, which is roughly the film absorbance at 600 nm.

Additionally, the sample transmittance, △*A*_T_, and reflectance, △*A*_R_, were calculated as shown in Figure 7b,c using the TFI model. This involved splitting the film in 100 layers and assigning to each layer the change of ε proportional to the local carrier density and assuming that two D-L parameters are affected; the lowest-energy Lorentz band amplitude, *A*_1_, and Drude amplitude *E*_D_, as explained previously. As can be expected, in the transmittance mode (Figure 7b) the strongest signal is the bleaching of the band gap absorption, which is virtually insensitive to the carrier distribution, though it is proportional to the total number of photocarriers. However, at other wavelengths, the response is sensitive to the carrier distribution and can be used to gain knowledge on photocarrier mobility in the film.

Another interesting outcome of the modeling is the change in the shape of the spectra as the distribution changes. Especially, in reflectance mode, one can notice a “shift” of the peak position close to 1000 nm and a flip of the signal from negative to positive at 1400 nm. It worth to emphasize that there is no any change in spectra of ε or ñ assigned to the photocarriers nor the number of the carriers, and only carrier positions are changing in time.

**Figure 8:**
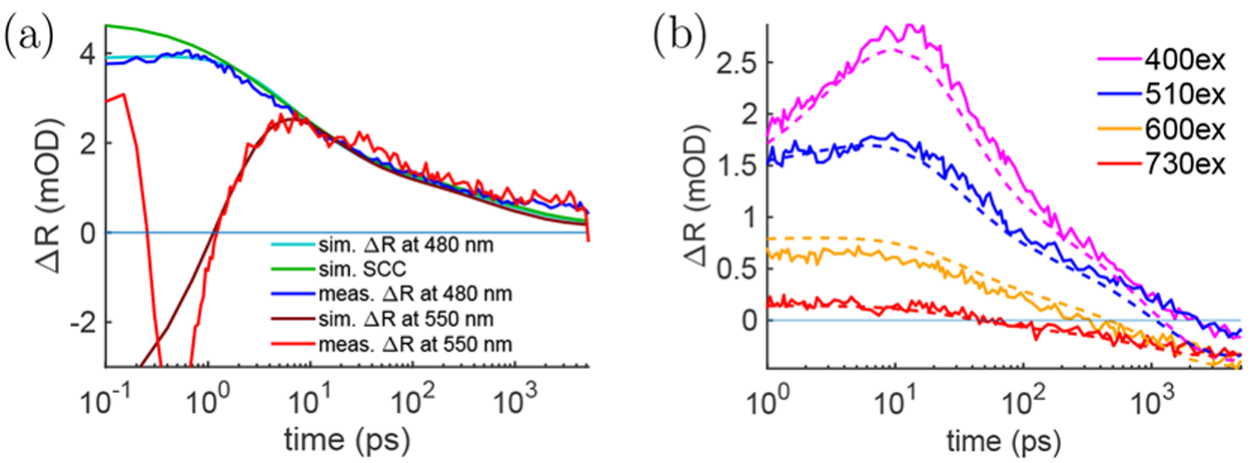
Measured and modeled CsMAFA perovskite TR signal (a) at 480 and 550 nm probes after excitation at 400 nm and (b) at 1125 nm probe after excitation at different pump wavelengths. In the TFI model the △ñ(λ) per carrier is kept the same, only the carrier distribution changes due to diffusion and recombination. By comparison, the SCC tracks the measured signal only after 1 to 10 ps depending on the wavelength. The signal at 550 nm was normalized to match the 480 nm signal at late delay times. Reprinted with permission from ref (58). Copyright (2021) Royal Society of Chemistry.

The TFI model used to calculate spectra in Figure 7b,c accounts for all the possible sources of interference by splitting the modeled film into multiple layers. In contrast, a commonly used approximation is to only consider the surface carrier concentration (SCC) and ignore potential interference effects if the film is opaque at the probe wavelength.^50,53,54^Figure 8a showcases a carrier mobility measurement for CsMAFA perovskite at visible wavelengths.^56^ Here, the TFI model is compared with the SCC model at two different probe wavelengths to showcase how the two models differ in accuracy. At both of the visible probe wavelengths, 480 and 550 nm, the perovskite layer is opaque enough to prevent light from passing through and being reflected from the other interface. However, there are differences between the measured signals and the SCC modeled response, which originates from the interference caused by the gradient in carrier distribution and refractive index. At 480 nm, the common lead-based perovskites have a TR signal that most optimally follows the SCC, whereas at 550 nm, the models only match after 10 ps. While the absorption coefficient is slightly larger at 480 nm than at 550 nm, the main difference stems from the different ratio between △*n* and △*k*. Another notable feature is how the carrier diffusion causes the TR signal to initially increase before it begins to decrease if excited by using a short pump wavelength, which can occur in both visible and NIR wavelengths.

Figure 8b shows a similar carrier mobility fit in the NIR region where the films are fully transparent. Here, the signals are initially positive yet turn negative at later stages. Such a response to carrier diffusion makes it easier to separate carrier diffusion from other effects such as carrier recombination. Similar diffusion-related signal changes have been reported for vanadium dioxide (VO_2_), which undergoes an insulator–metal phase transition when its temperature is close to or over 68 °C.^34,61,62^ Lysenko et al. recognized that the TR signal is dependent on the film thickness due to TFI and heat diffusion.^34^ Madaras et al. modeled the TR signal of VO_2_ as layers of metal and insulator phases where the transition to metal phase spreads in pico- and nanosecond time scale after photoexcitation.^61^

There have also been numerous time-resolved differential reflectivity studies on various transition-metal dichalcogenide monolayers or thin exfoliated flakes,^63−67^ or van der Waals heterostructures.^68−72^ These samples were typically deposited on opaque silicon wafers, and thus the pump–probe reflectance mode was used for estimating carrier lifetimes and photophysics. For these samples, when the one-dimensional diffusion presented in Figure 8 is not an issue, either because (a) the diffusion is very slow compared to carrier lifetime or (b) the sample has low absorption at the pump wavelength, then the decay of TR signal can be evaluated as usual, similar to eq 1. In many cases the carrier diffusion was measured by mapping the spread of carriers spatially in two dimensions,^73−76^ where similar care should be taken to minimize diffusion in the third dimension.

## Interfacial Phenomena and Charge Transfer

**Figure 9:**
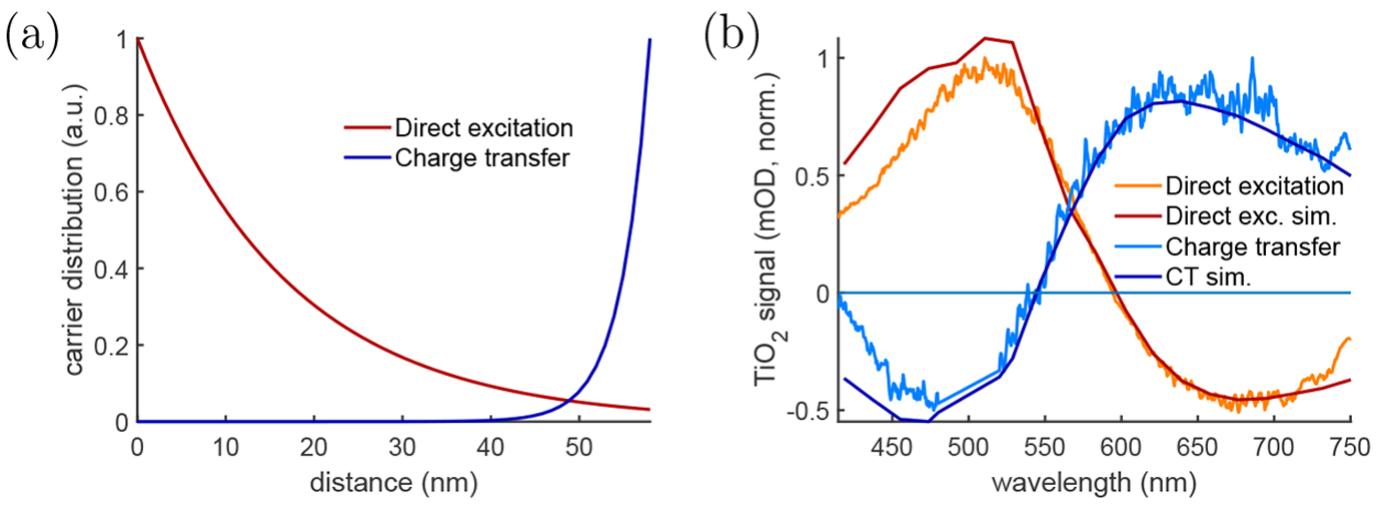
(a) Charge carrier distribution in TiO_2_ thin film after excitation at 320 nm (red) or after charge transfer from underlying silicon (blue) and (b) the corresponding measured and modeled TR signals. Reprinted with permission from ref (60). Copyright (2023) Elsevier BV.

Another case where carrier distribution can make a significant difference is charge transfer studies. Khan et al. reported an example where a 58 nm thick layer of TiO_2_ was deposited on silicon.^60^ The TiO_2_ can be excited directly using a 320 nm pump, with carrier distribution shown by the red curve in Figure 9a. The corresponding measured TR signal is also presented by the red curve in Figure 9b with the wavy shape caused by thin film interference. However, if the underlying silicon is excited instead (for instance, by using a 500 nm pump), there is charge transfer from Si to TiO_2_ which produces an opposite signal (the blue curve in Figure 9b). By modeling the signal from transferred carriers at the TiO_2_–Si interface, it was possible to reproduce the measured charge transfer signal and show that charge accumulation at the surface can lead to reduction in further charge transfer. This demonstrated that the TR signals can be very different if the charge carriers are generated at opposite interfaces, and TR responses have to be modeled on a case-by-case basis to understand the underlying phenomena.

Although charge transfer from the active layer to the hole or electron acceptors is one of the most common and relevant research topics for perovskites and other photoactive materials, transient reflectance has seen very little use in this field despite its potential to separate surface and bulk phenomena. Attempts have been made to measure charge transfer between perovskite and various electron transport layers,^49,77,78^ but these studies focused on the perovskite signal at the band gap to measure carrier diffusion instead of attempting to find a signal from the charge acceptor layer itself with the help of TFI modeling.

Aside from carrier diffusion studies, transient reflectance has been utilized for studying the effects of interfacial 2D passivation on perovskites^79,80^ or surface recombination and diffusion.^53,54,57,59,81,82^ The time-resolved perovskite charge transfer studies have had two major obstacles: first, lead halide perovskites tend to have orders of magnitude stronger signal than the acceptor layers, and second, the measurement range has been limited because of high absorption in the visible range and challenges of interpreting the TFI signal in the NIR. Thus, we hope that the methods and models provided in this article enable much more sophisticated studies across the entire spectral range for charge transfer and other relevant phenomena.

All examples provided above presume homogeneous monolayer films. Typical samples of interest may consist of a few layers. For example, in perovskite solar cells, the perovskite film is placed between electron and hole transporting layers. Another example is a TiO_2_ layer on top of silicon as discussed above. Optical properties of such samples can be calculated by dividing the sample into homogeneous layers and using TMM to account for TFI in such films. This requires knowledge of each layer thickness and complex refractive index *ñ* = *n* + *ik*. However, only two independent values are available from the TAS measurements, △*T* and △*R*, and only for homogeneous monolayer film (△*T*, △*R*) can these be recalculated to (△*n*, △*k*) for known film thickness, as discussed above. There is no general solution for multilayer films since the number of unknown variables exceeds the number of measured values. However, if all but only two parameters affecting the optical properties of the sample are known, the two unknown parameters can be extracted from TAS measurements. For example, analysis of carrier diffusion discussed above was based on the knowledge of initial carrier distribution and the assumption that a simple 1D diffusion equation can describe the carrier migration across the sample after photoexcitation.^55^

Sample inhomogeneity presents another potential limitation of the analysis approach discussed here, particularly evident in domain-like structures typical for perovskite and TiO_2_ films.^36,56^ If the characteristic scale of lateral inhomogeniety is much smaller than the monitoring wavelength, the inhomogeneity has no significant effect on the measured TAS response. However, inhomogeniety that leads to uncertainty in the film thickness may become more critical as it starts to affect TAS and produces a period of wavy shaped spectra (Figure 1c). Even a few nanometer differences may have a detectable impact on the measured outcome. Nonetheless, if the scale of surface roughness is smaller than the monitoring wavelength, the effect of roughness can be accounted for by splitting the rough region into multiple thin layers with properties smoothly transitioning from the surface to the film bulk material.^55^

## Conclusions

Although transient absorption spectroscopy (TAS) is a widely spread experimental technique with straightforward to use commercially available instruments, interpretation of the measured results needs some care and may not be so straightforward as it seems at the first glance. The focus for this paper is TAS of thin films and, more precisely, the effect of the photoinduced change in refractive index. Although the standard TAS transmittance response will originate from the photoinduced absorbance change at wavelengths of high film absorbance, there are conditions when the observed signals will be determined by the change in refractive index instead. The problem can be identified by measuring both the transmitted and reflected probe light, which can be used to separate the effects of the photoinduced change of refractive index and absorption coefficient. Furthermore, specifically to thin films, the thin film interference complicates the analysis for the spectral shape of the photoinduced response, and additional calculations of the interference may be needed.

Despite the obvious complications imposed by the photoinduced change of refractive index, this phenomenon opens new opportunities in photocarrier dynamics studies. The carrier diffusion across the film was already studied using TAS and proved to be unique in monitoring charge migration on the nanometer scale and picosecond time domain. Also, transient reflectance can be used with nontransparent samples, which greatly expands applicability of the TAS technique. However, precise modeling requires good understanding of the factors affecting reflectance, such as the carrier distribution and compounding effects of photoinduced changes in both absorption and refractive index.
